# 629. High Efficacy of Bictegravir/Emtricitabine/Tenofovir Alafenamide (B/F/TAF) in African American Adults with HIV Including Those with Preexisting Resistance, Viral Blips, and Suboptimal Adherence

**DOI:** 10.1093/ofid/ofab466.827

**Published:** 2021-12-04

**Authors:** Kristen Andreatta, Michelle L D'Antoni, Silvia Chang, Aiyappa Parvangada, Ross Martin, Christiana Blair, Sean E Collins, Kirsten L White

**Affiliations:** 1 Gilead Sciences, Inc, Foster City, CA; 2 Employee, San Mateo, CA

## Abstract

**Background:**

BRAAVE 2020 demonstrated the efficacy of switching to bictegravir/emtricitabine/tenofovir alafenamide (B/F/TAF) among African American adults with suppressed HIV through Week (W) 48 (Figure 1). We present resistance, viral blips, adherence, and virologic outcomes through W72.

Figure 1. BRAAVE 2020 study design (phase 3, randomized, open-label, multicenter [USA], active-controlled study) and virologic suppression at weeks 24 and 48

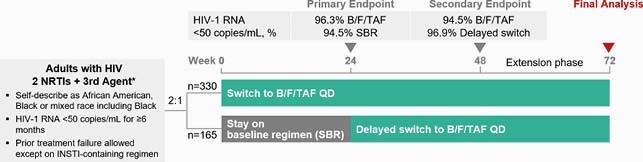

*Allowed 3rd agents: any FDA-approved protease inhibitor, nonnucleoside reverse transcriptase inhibitor (except etravirine), integrase strand transfer inhibitor (except bictegravir), or maraviroc.

**Methods:**

Enrollment criteria permitted NNRTI resistance (-R), PI-R, and certain NRTI-R (M184V/I allowed; K65R/E/N, ≥3 thymidine analog mutations [TAMs], or T69-insertions excluded) and excluded known primary INSTI-R. Preexisting drug resistance was assessed with historical genotypes and retrospective baseline proviral DNA genotyping. Adherence was calculated by pill count. Viral blips (transient HIV-1 RNA ≥50 copies/mL) and outcomes based on last available on-treatment HIV-1 RNA were assessed.

**Results:**

489 participants received B/F/TAF and had ≥1 post-switch HIV-1 RNA measurement. Baseline genotypic data from cumulative historical and/or proviral genotypes were available for 96% (468/489) in protease/reverse transcriptase and 93% (453/489) in integrase. Preexisting NRTI-R, M184V/I, ≥1 TAMs, NNRTI-R, and PI-R were observed in 15% (68/468), 11% (50/468), 8% (36/468), 22% (101/468), and 13% (61/468), respectively. Primary INSTI-R was detected post-randomization in 2% (11/453); all remained in the study and were included in efficacy analyses. Through W72, 99% (486/489) of participants had HIV-1 RNA < 50 copies/mL at their last study visit, including all with baseline NRTI-R or INSTI-R (Figure 2). Mean frequency of viral blips was 1% per timepoint, and blips were not associated with virologic failure. 112 participants (23%) had < 95% adherence by pill count, 98% (110/112) of whom had HIV-1 RNA < 50 copies/mL at last visit, including 14 of 14 (100%) with < 80% adherence. No participant discontinued due to lack of efficacy or had treatment emergent resistance to study drugs.

Figure 2. Virologic suppression by preexisting resistance, viral blips, and adherence

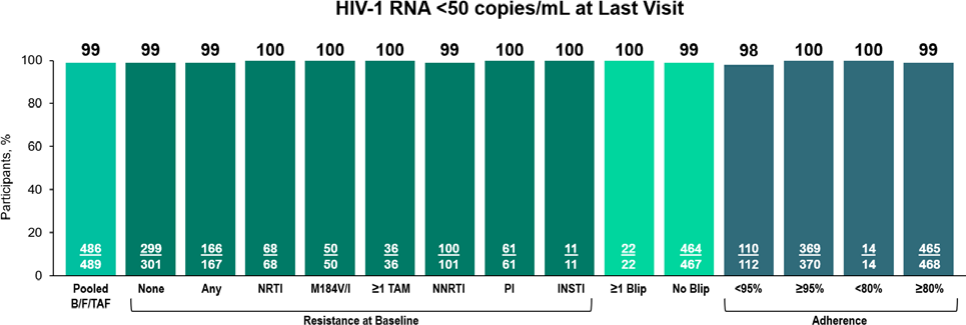

**Conclusion:**

Virologic suppression was maintained through W72 of B/F/TAF treatment, including those with preexisting resistance, viral blips, and suboptimal adherence. Continued HIV suppression and absence of treatment-emergent resistance demonstrate the efficacy of B/F/TAF in African Americans regardless of adherence or preexisting resistance to NNRTIs, PIs, or non-tenofovir NRTIs.

**Disclosures:**

**Kristen Andreatta, MSc**, **Gilead Sciences, Inc** (Employee, Shareholder) **Michelle L. D'Antoni, PhD**, **Gilead Sciences** (Employee, Shareholder)**Gilead Sciences, Inc** (Employee, Shareholder) **Silvia Chang, Masters**, **Gilead Sciences, Inc** (Employee, Shareholder) **Aiyappa Parvangada, MS Computational Biology**, **Gilead Sciences, Inc** (Employee, Shareholder) **Ross Martin, PhD**, **Gilead Sciences, Inc** (Employee, Shareholder) **Christiana Blair, MS**, **Gilead Sciences, Inc** (Employee, Shareholder) **Sean E. Collins, MD, MS**, **Gilead Sciences, Inc** (Employee, Shareholder) **Kirsten L. White, PhD**, **Gilead Sciences, Inc** (Employee, Shareholder)

